# Bibliography

**Published:** 1901-02

**Authors:** 


### Bibliography.

STUDENTS' EDITION. A PRACTICAL TREATISE ON MATERIA MEDICA AND THERAPEUTICS, WITH ESPECIAL REFERENCE TO THE CLINICAL APPLICATION OF DRUGS. By John V. Shoemaker, M.D., LL.D., Professor of Materia Medica, Pharmacology, Therapeutics, and Clinical Medicine, and Clinical Professor of Diseases of the Skin in the Medico-Chirurgical College of Philadelphia, etc., etc. Fifth Edition, thoroughly revised. T. A. Davis Company, Publishers, Philadelphia, 1900.

The author in the preface to this edition, says of it, "The experience of the author in the class-room has led him to make a change in the scope of the fifth edition. So many new remedies from the chemical laboratory and from the vegetable kingdom have been introduced during recent years that he had decided to divide the work into two independent issues, one (the present) to be known as the Students' Edition and the other, which will be forthcoming shortly, as the Physicians' Edition." This is certainly a wise discrimination, for most works on materia medica and therapeutics, where intended for both classes, fail to satisfy either, for with the student they cover too much ground, and for the practitioner they are not sufficiently extended.

Part I. concerns itself with "General Considerations concerning Remedies and Systems of Therapeutics," and exhibits such a liberal spirit that the author's opinions are worth quoting. "In order to avoid misapprehension, it may be proper at the outset to explain that in the present treatise this system of scientific, or so-called 'regular,' medicine will be followed. Scientific, or regular, medicine is quite distinct from any school or sect in medicine, and is equally separate from so-called 'allopathy' or 'allopathic practice.' As every educated physician knows, there is a radical difference between 'an allopathic doctor' and a 'regular practitioner,' inasmuch as one is sectarian and the other non-sectarian. In point of fact, at the present day there are no allopathic physicians, and, of course, no 'allopathic' examining boards, and all followers of scientific medicine should resent the application of such a sectarian title to designate the regular practice of medicine.

"At the same time that we discard restrictions as to therapeutics and claim the right to employ whatever remedial means experiment and observation lead us to believe will benefit our patients, it should not be forgotten that the knowledge at our command is derived from various sources, and if we are willing to acknowledge the indebtedness of modern medicine even to native tribes for many useful remedies, we should not be above admitting the fact that useful lessons may also be occasionally learned from followers of exclusive schools of medicine, or so-called irregular physicians. 'Every judicious physician,' says Dunglison, 'must be an eclectic' in the sense that he selects from every source the best means of controlling disease." Whether this broad foundation will suit all classes of medical thought is doubtful, but that it is a true position to take accords with that advanced therapeutics that appeals to liberal practitioners. It is difficult to understand why any method of treatment should be rejected simply because of its origin, or why credit should not be given, even though that may bring some degree of credit to what may be regarded as empirical practice. When therapeutics has a basis upon absolute science, it may then be possible to repudiate those methods that have a somewhat discreditable origin, but until then everything should be used that corresponds with good judgment.

Considerable space is properly given to "Pharmacy." This is followed by "Prescription Writing and Formulæ." This is clearly written, and the student should not experience difficulty in following the author. In this chapter and throughout the book the metric system is given the preference, hence in all formulæ the prescription begins with the amount to be used in this system, with the equivalent in apothecaries' weight. This materially assists in bridging over a difficulty experienced by all in transferring the thought from one to the other, especially when long habit has made change from one system to the other one requiring considerable study and practice.

This book has not been prepared to meet the needs of dental students, and hence the therapeutics necessary for these will not be found upon its pages, but, when intelligently trained, these should have no difficulty in making proper use of agents described. It is noticeable, however, that when the author steps aside to give any opinion upon dental matters, which he rarely does, he seems to fail to measure to his generally good standard. For instance, we find him stating that "Caries of the teeth may be relieved by a mouth-wash containing carbolic acid or phénol-sodique, well diluted." Dental patients and, at times, dental practitioners, would be delighted if caries could be benefited by this simple process, but that time will never arrive through the use of any mouth-wash.

In the article on "Barii Dioxidum" it is stated that hydrogen dioxide, "when pure and of official strength, is free from irritating qualities, and can be poured over wounds, injected into sinuses or into the ear, or used as a spray in ulcerations of the pharynx and larynx." This seems to the reviewer to be given altogether too wide a use for this agent, and without qualification would lead the student to regard it as a very harmless agent, whereas, if injected into cavities with small outlet there may be produced serious harm, and then it is very difficult to keep hydrogen dioxide down to proper strength. Those who have had extended experience with it have an increasing dread of using it as furnished by the manufacturers. The reviewer has observed quite serious results following its use without care in noting percentage in strength.

Again we find the author falling into an error, wherein he says, "It [hydrogen dioxide] enables the dentist to treat and fill, at the same sitting, a sensitive pulp or cavity." Exactly how a sensitive pulp can be filled has not yet been discovered by the average dentist. It is presumed, however, the author means the pulp-canal, and this is probably a typographical error; but the use of this agent does not enable the dentist to fill that canal at one sitting, although, if properly used, it is one of the best antiseptics to effect that object in, possibly, repeated sittings.

In the article on "Cinchona" the author says of quinine, "A one per cent. solution of quinine sulphate is recommended as a topical treatment of sluggish, unhealthy, infected wounds." This entirely coincides with the reviewer's experience, as he has used it with decided results in the treatment of pyorrhœa alveolaris.

Under the head of "Naphthol," the author expresses very positive opinions regarding this agent. This accords with his earlier published views, as he was one of the first to recommend it as one of the best and safest antiseptics. He says of it here, "It is one of the most powerful antiseptic agents, possessing three times the strength of carbolic acid or iodoform and four times that of creosote or naphthalin. It may be regarded as absolutely safe, since, according to Professor Bouchard's investigations, nearly half a pound would be required to cause death in a healthy person weighing one hundred and fifty pounds." The author does not regard hydronaphthol anything more than "an impure form of beta-naphthol."

It is unnecessary to follow this interesting and valuable publication throughout. Arranged to meet the wants of the medical student, it should be to him an invaluable aid, and doubtless this has been the experience, as the demand for a fifth edition testifies more strongly than words.

A Practical Treatise on Artificial Crown- and Bridge-Work and Porcelain Dental Art. By George Evans, Lecturer on Crown- and Bridge-Work in the Baltimore College of Dental Surgery, etc. Sixth Edition revised. With six hundred and fifty-one Illustrations. The S. S. White Dental Manufacturing Company, Philadelphia, 1900.

It would almost seem an unncessary duty to review this well-known and well-appreciated work, for the fact that it has reached a sixth edition is quite sufficient testimony to the estimate in which it is held by dentists everywhere.

In the preface to this edition the author expresses himself in no uncertain terms in regard to one of the worst features of crown- and bridge-work,—that of mutilating teeth in order to place crowns, when under older methods these could have been filled. The temptation to crown has been, in the opinion of the reviewer, one of the debasing results of inartistic modern dentistry. The author says, "Practitioners of to-day seek methods which will permit the attainment of the desired results in crown- and bridge-work without the devitalization of pulps, with the least possible mutilation of the natural teeth and the smallest exposure of metal, and which are least complex in construction. These ideas have largely influenced the conduct of the revision of this edition."

There is, probably, no branch of dentistry that has shown greater progress than this of crown- and bridge-work. The present practitioner of dentistry does not have to be a very old man to remember when a crown was an unknown quantity in modern dentistry, but, if we are to credit history, it was not unknown to ancient civilizations. Although so few years have elapsed since crown introduction, we have this book of Dr. Evans, a pioneer in the specialty, covering the ground so thoroughly that it would seem impossible to make any valuable additions to the mechanical construction. Whether the dentistry of the future will tolerate bridge-work remains for time to determine. There seems to be a growing sentiment in opposition to it. Its mutilation of teeth and the contamination of the breath in permanent bridges being factors in causing much opposition.

The author very properly objects to the indiscriminate devitalization of pulps, for he says, "An examination of the investing membranes of pulpless teeth, as treated generally, shows the existence of a percentage of abnormal conditions, by which their firmness is to some extent impaired, their susceptibility to acute inflammation increased, and their reliability as foundations for crown- and bridge-work greatly lessened when compared with teeth which have living pulps." There can be no question as to the truth of this.

The first part of the book is devoted to treatment, and the real subject-matter, that of crowns, begins with Part II., "Artificial Crown-Work." The various crowns in use are here thoroughly described with methods of manufacture. There should be no difficulty in following the instructions of the author. The very perfect illustrations make any doubtful points in the text perfectly clear. A very moderate degree of knowledge in the working of metal should enable the beginner to succeed by closely following the minutely detailed explanations.

Part III. opens with "Bridge-Work." The author is as careful here as he has been in previous chapters to give all well-known methods. The student of this branch has, therefore, no need to wade through much of the literature of the subject, but will find all methods clearly explained and illustrated. In some respects the author's tendency to a fair exposition of the work of others leads him to describe and illustrate specimens of bridge-work that should, in the reviewer's opinion, never be placed in the mouth. In the earlier period of this branch of practice, extremists placed entire dentures on, possibly, four roots. That this was wrong practice has been amply demonstrated by experience. The author advocates the sectional system.

Porcelain inlays have assumed increased importance of recent years, and the author devotes a chapter to "Porcelain Dental Art," in which he includes Dr. N. S. Jenkins's "Low-Fusing Porcelain" and method of working, as well as others of recent date.

The book, as presented in this edition, is certainly one of the most valuable additions to the literature of this portion of prosthetic dentistry produced at the close of the century. While there is a natural aversion to bridge-work in the minds of many operators, in which the reviewer shares a degree of sympathy, it must be said of it that it saved mechanical dentistry from utter ruin through the introduction of rubber as a base for artificial teeth. From that period the working of metal became almost a lost art in dentistry. Crown- and bridge-work necessitated a high degree of mechanical skill, and obliged dentists to acquire ability in this direction or suffer loss in practice. To Dr. Evans is due a large share of credit for the rehabilitation of this skill in metal working.

To those who need this instruction and those who have already acquired skill in this work, this book will be of great service, as one to which constant reference may be made with satisfactory returns for the labor given.

The work is rich in illustrations and entirely satisfactory in general make up.

PRINCIPLES AND PRACTICE OF FILLING TEETH. By C. N. Johnson, M.A., L.D.S., D.D.S., Professor of Operative Dentistry in the Chicago College of Dental Surgery. With Illustrations. The S. S. White Dental Manufacturing Company, Philadelphia; Claudius Ash & Sons, London, 1900.

The author of this book evidently felt when preparing it that a work on operative dentistry was not a crying need, for he says, in his introductory, "The thought of the profession in recent years has been too active along those lines for any one individual to claim much in the way of originality."

The book, very properly, opens, Chapter I., with "Deposits on the Teeth." This is the orderly way of beginning operations upon the mouth. First, free the teeth and gums of all deposits, for, as the author states it, "It matters not how beautiful or how perfect an operation may be under these conditions, the work should never be considered as ideal service."

The first thirty pages are taken up with this subject, not too much when its importance is considered. This is followed by "Dental Caries," in which the author simply follows Black and Williams, and has very little to say of Miller's work. He gives Black credit for "calling attention to the fact that this acid (micro-organic product, Miller) must be formed and allowed to act immediately at the point where the decay was to begin." This discovery, if it be such, does not belong to any modern writer, for it is almost as old as dentistry. The point of impact with resulting acid was always understood. This loose way of writing history needs correction.

The author assumes, with Black, that the gelatinous plaque of Williams has been proved to be the product of micro-organisms, and that under or through this, the organisms produce the acid immediately destructive to the enamel. This has never been proved by Williams, or any one else, so far as the reviewer is aware, remaining simply a plausible theory.

The preliminary operations for filling are well described, but the author evidently has not had much practical experience with napkins used to prevent moisture entering the cavity. Skill in this direction belonged to an earlier generation, and is practically a lost art with present operators.

In the "Classification and Preparation of Cavities," we have presented the "extension for prevention" idea of Black. The author says of this, "If, in the preparation of a cavity, we limit the area to a small round outline, we have left unprotected, at the points indicated, more or less of the surface of enamel. With the same conditions present and the same influences at work which originally induced decay, there is little to prevent a recurrence. The remedy lies in so extending the outlines of the cavity that the margins are carried to a point where they will be kept clear.

This process has been termed 'extension for prevention' by Dr. G. V. Black, and its observance must be insisted upon where the most permanent work is required." To demonstrate this the author gives three illustrations. The first carried the extension to the gingival border in diagrammatic lines. If this be the explanation of extension for prevention, the less the dental profession has of it the better. If the reviewer understands it, a pin-head cavity on a proximal surface of an incisor must be enlarged until it extends to, or under, the gum border. This means placing the upper border of the cavity at a point where it will be constantly endangered by acid secretions. The author later on gives a reason for this curious operation, which is even more fallacious than the operation itself. He says, "The reason for this extension is the well-known fact that wherever we have the gingival portion of a perfectly inserted filling, covered by healthy gum-tissue, we will never have recurrence of decay at that point." Again he says, "No tooth may be considered safe from recurrence of decay around proximal fillings unless the gingival wall has been covered sufficiently root wise to bring that portion of the filling under the gum."

In the treatment of other proximal cavities the author says of a certain class that the "rule should be to open the cavity to the occlusal surface." In other words, if the cavity presents on the proximal surface of a premolar or molar tooth, the dentist should not rest satisfied with preparing the cavity as it stands, but must cut away and make a cavity on the occlusal surface. Such advice is not only contrary to accepted methods, but is radically wrong from every point of view except that of extension for prevention.

In the formation of cavities preparatory to inserting fillings, the author has this to say: "As the gingival wall joins the buccal or lingual wall, it should form a distinct angle in the axial region, but should execute a short curve at the enamel margin. . . . When shaped, as first outlined, it affords a base upon which the filling may be built without danger of the gold rocking under the plugger-point." This, as described on paper is an excellent theoretical form, and if always possible would be a solution of a difficulty, but teeth do not always decay to suit the dentist, and he is forced to prepare the cavity for each individual case, a filling properly anchored should never rock; in fact, the first piece of gold should be as stable as the last, and this is quite possible of attainment, possibly by the author's method, and certainly by other systems.

The author has not much faith in cataphoresis or other methods for reducing sensitive dentine, and sums up the whole question by stating that the management of this " resolves itself to the following summary: Manipulative skill on the part of the operator, tact in knowing how to control the different temperaments among our patients, and the invariable use of the keenest, sharpest instruments." This is not very encouraging, but it contains many grains of truth.

The author does not regard the conductivity of gold as a deterrent factor in its use. He says, " Gold has frequently been severely censured when the brief factor at fault in the case has been the presence in the cavity of a hypersensitive mass of decalcified tissue, which should have been removed in the preparation of the cavity." This constitutes a serious arraignment of every operator who has made this charge against gold, and the number is legion. It is not true that masses of decalcified dentine are generally left in the cavity, and it is equally untrue that this mass is necessary to carry sensation to the pulp. Such a statement seems to imply a need of more intimate knowledge regarding the histological features of tooth tissues.

The author devotes but a half-page to the consideration of tin as a filling-material, and evidently is not practically familiar with the best methods of working it. This is unfortunate, for it has a place hardly second to any other material properly used and properly placed.

Inlays are not regarded with much favor. They are held to be " ephemeral" and not worthy to be classed with that work, " the result of hard, painstaking effort on the part of our illustrious predecessors."

In the use of mallet force the author has this to say: " One very great advantage of the softer mallets is that a much heavier mallet can be tolerated by the patient than if made of steel, and the condensing power is thereby materially increased without discomfort." This is a very great error. The heavy lead mallets do not increase condensing power. They lessen it; or, in other words, they increase mobility or motion by the increase of weight. The mallet that will overcome this moving force will produce greater solidity at the point of impact. The reviewer demonstrated this thirty years ago. (*Dental Times.*)

The author's description of the amount of arsenic to be used to devitalize a pulp is not correct, and it is also not a scientific way of stating it: "A minute quantity, one-half or even one-fourth the size of the head of an ordinary pin, . . . will be found ample for its destruction." This is certainly very much more arsenic than necessary for the devitalization of a single pulp.

The advice given to "wait a week or ten days before removing the pulp" is bad. It requires about that period for decomposition to take place, and then pericemental inflammation is almost a certain result.

In the treatment of fistulæ the author adopts a curious method: "The root-canal should be filled and the external fistula packed with cotton to enlarge it. The cotton should be changed every twenty-four hours for a larger piece till the fistula is sufficiently expanded to permit of perfect access to the end of the root. When this is attained, a sharp bur in the engine should be used to ream out the carious bone and smooth the rough end of the root." The operation is a good one so far as the removing necrosed tissue is concerned, but why delay matters with cotton pressure when the knife or properly prepared instruments could more readily and immediately remove the intervening tissue?

The author, following Black's dictum, that metal can be used at any age, says, "It is not a question of age at all. It is a question of temperament; a question of physical and mental stamina on the part of the patient." This is a very remarkable statement, coming from the source it does, and is unworthy the author's recognized intelligence.

It is not pleasant to be forced to select here and there a statement which seems to controvert accepted methods or recognized facts, but this is not an ordinary book. Operative dentistry lies at the very foundation of all dentistry, and the author and critic must draw closely to the line of accepted practice. A book of this character is supposed to teach the rising dental generation how to treat teeth, and especially how to fill cavities caused by inroads of caries. It should, therefore, be entirely practical, and must be judged from that stand-point and that alone.

Readers of this book must admire the spirit of the author, manifested in every line, that of an unselfish devotion to the highest ideals. It is not his fault if he fails to inspire a similar feeling in those who follow him. In the sense of an inspiration to good work the book can be cordially recommended, but it is hoped, if it reaches a second edition, that the author will be able to take a broader view of operative dentistry than he seems capable of doing at present.

The work is very satisfactory in its typographical make-up and in its illustrations, a very usual feature of all the publications of the S. S. White Dental Manufacturing Company.

